# From sole crops to strip cropping: Decision rules of frontrunner farmers in The Netherlands

**DOI:** 10.1371/journal.pone.0329133

**Published:** 2025-07-24

**Authors:** Stella D. Juventia, Dirk F. van Apeldoorn, Hilde Faber, Walter A. H. Rossing

**Affiliations:** 1 Farming Systems Ecology Group, Wageningen University & Research, Wageningen, The Netherlands; 2 Field Crops, Wageningen University & Research, Edelhertweg 10, Lelystad, The Netherlands; 3 Centre for Crop Systems Analysis, Wageningen University & Research, Wageningen, The Netherlands; 4 Land & Co, Costerweg, Wageningen, The Netherlands; Canakkale Onsekiz Mart University, TÜRKIYE

## Abstract

Strip cropping, where several crops are grown in adjacent long and narrow multi-row strips, is an innovation niche that challenges monocropping by offering a greater range of ecosystem services, including higher biodiversity and aesthetic value at similar yield. It can be implemented within the current regime by adjusting the strip width to fit machinery working width. However, its novelty and complexity, that mobilize four dimensions of diversity—space, time, gene, and operational crop management—make transitions from monocropping difficult. This study aims to learn from the experiences of strip cropping frontrunners by: 1) capturing the contexts, objectives, challenges, and outcomes of farmers’ first-year strip cropping experience, and 2) identifying patterns in farmers’ decision rules following its uptake. Semi-structured in-depth interviews were conducted with ten Dutch farmers with at least one-year strip cropping experience. Upon formulating the farmers’ operational management decision rules, we used two analytical lenses to find patterns in the changes compared to monocropping. Results showed that all farmers shared the objective of increasing insect biodiversity. Common challenges included a lack of agro-ecological knowledge and experience, incompatible machinery working width, and crop neighbor damage. Most farmers positively evaluated the feasibility to adjust or acquire adapted machines, were neutral on yield changes, and negatively evaluated workload. We identified 49 decision rules comprising 113 condition-decision relations. We found two clusters or archetypes of farmers that differed in their propensity to adjust mechanization. No pattern was found among the other adjustments from monocropping to strip cropping, indicating that changes were highly farmer-specific. The two most often mentioned decisions included machine investment and crop choice adjustment. These apparent key decisions may guide exchanges among strip cropping farmers, advisors, and researchers. Leveraging diverse decision rules captured in this study, alongside strengthening the infrastructure and institutional support for strip cropping will help farmers transition towards sustainable agricultural systems.

## 1. Introduction

While modern industrial agriculture has increased food production to levels that could theoretically sustain the current global population [1], it has done so through intensive use of synthetic fertilizers and pesticides and by focusing on a narrow selection of high-yielding crops and varieties [2]. This approach has led to environmental degradation, transgressing the planetary boundaries [3–6]. Two broad narratives dominate the quest for farming practices that can maintain food production while positively impacting the environment and securing farmers’ livelihood [7–9]. The first narrative revolves around the promise of innovations within the dominant agricultural model to achieve higher efficiency and lower negative externalities. This narrative is often associated with the digitalization of agriculture through, for example, precision farming [10–12]. The second narrative features radical innovations from outside the dominant agricultural model through transformative redesign of farming systems based on agroecological principles [13]. It is associated with organic farming and a wide range of other visions linked to enhancement of supporting and regulating ecosystem services, reduction of external inputs, and provision of equal or more nutritious food compared to conventional systems [14,15]. While radical innovations may come from outside the dominant agricultural regime, they can also develop in what have been referred to as hybrid innovation niches, i.e., places within the dominant regime where transformative ideas take shape [16–18]. An example of a hybrid niche is strip cropping.

Strip cropping challenges the existing regime, which is based on sole-crop monoculture by putting forward within-field crop diversification as an alternative strategy [18]. Strip cropping systems can be designed in such a way that they can operate largely within the confines of current mechanization, rules and regulations, and markets [19]. Strip cropping is a form of intercropping where several crops are grown adjacent to one another in long and narrow multi-row strips, making it an operable practice by existing machines [19–21]. It offers potential for delivering a greater range of ecosystem services compared to sole-crop monoculture by mobilizing the three dimensions of diversity at the field level: spatial, temporal, and genetic diversity [4,21]. Spatial diversity is created through alternating patterns of multiple crops within a field at a given point of time; temporal diversity through crop rotation and relay intercropping; and genetic diversity through the different crops in the strips and cultivar mixtures within individual strips [21]. Research has shown the potential of strip cropping systems compared to sole-crop monoculture in terms of enhanced biodiversity, pest and disease suppression [see, e.g., 21, 22, 23], and yield [see, e.g., 24, 25, 26]. From a management perspective, strip cropping offers potential compared to other types of intercropping, such as row intercropping where two species are cultivated in alternating rows, as the strip width can be adjusted to fit the working width of current mechanization. In Europe, a strip width of three or six meters can be implemented in strip cropping systems given the current machines and their working widths [27]. This could negate the need to invest in new machinery for farm operations, one of the barriers that hinders farmers from diversifying their cropping systems [28,29].

Despite the potential benefits of strip cropping, it is as yet neither widely adopted nor well-studied in the European context where most intercrop systems concern mixed intercropping, i.e., two or more species cultivated in the same field without a distinct pattern [21,24]. In general, lack of technical knowledge and increased management complexity are challenges practitioners face when adopting crop diversification practices [18,30]. In the context of strip cropping, the novelty and complexity of strip cropping systems design, which introduces the concept of spatio-temporal configurations, makes the transition from sole-crop monoculture difficult [19]. Building on the knowledge acquired by frontrunner farmers who already experimented with strip cropping would facilitate transitions to take place, although the extent of a wider systemic change also depends on institutional support and societal developments beyond strip cropping actors’ control [31].

Within Europe, strip cropping began to develop in the Netherlands, where strip cropping has been proposed as a strategy to contribute to the government’s vision on ‘Transition to a sustainable food system’ [32]. At the time of writing, knowledge on strip cropping was exchanged within a frontrunner farmer network that emerged following workshops by researchers for advisors and aspiring farmers [19]. Despite the emerging institutional support (e.g., subsidies for eco-schemes [33]) and societal enthusiasm over the visual appearance of strip cropping, widespread uptake was limited. Among the reasons, farmers mentioned marketing opportunities and implementation problems [19]. A couple of frontrunner farmers adopted strip cropping autonomously with considerable risk-acceptance, while the majority had reservations. Here we aim to learn from these frontrunner farmers on how they experienced strip cropping and made decisions at farm level, the lowest management level where transformation originates [34], when implementing strip cropping plans during their first year of transitioning from sole-crop monoculture. We do so by tapping into the flow of unformalized ‘unspoken knowledge’ of frontrunner farmers that usually remains implicit and is rarely recorded on paper as advisers or researchers rarely ask about them [35,36] for the purpose of supporting wider implementation of strip cropping systems.

According to Merot *et al.* [37], analyzing the implementation of novel farming practices would require understanding of farmers’ contexts, production goals, constraints, planning of farm practices, and decision-making rules. Therefore, two research objectives were formulated: 1) to capture the contexts, objectives, implementation challenges, and outcomes of farmers’ first-year experience with strip cropping implementation, and 2) to identify patterns in frontrunner farmers’ decisions following uptake of strip cropping. Addressing these research objectives is expected to assist aspiring and current strip cropping farmers, advisors, and researchers in the design and development of farm-specific strip cropping systems.

In the next sections we describe the procedures for data collection and analysis. In the results section, we present farmers’ contexts, objectives, challenges, and outcomes. We abstract farmers’ decision rules and visualize them in the form of a decision tree and a cognitive map. We then identify patterns in farmers’ decision rules using two analytical lenses. Finally, we discuss the implication and applicability of the knowledge acquired by this study to facilitate research and wider implementation of strip cropping.

## 2. Materials and methods

### 2.1. Data collection

**Fig 1:**
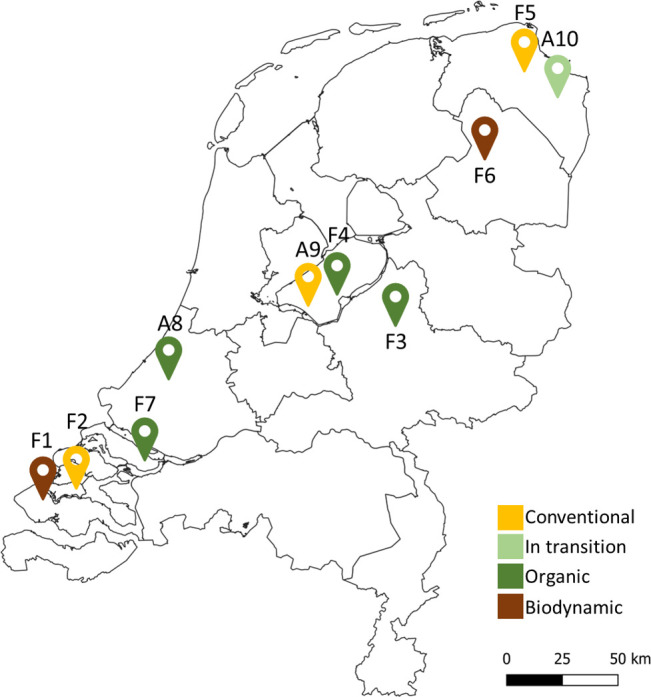
Locations of the seven (F1-F7) farmers and three additional farmers (A8-A10) interviewed in 2021. Map adapted from CBS Gebiedsindelingen 2024 WFS, via www.pdok.nl.

In the early stage of strip cropping development in the Netherlands in 2021, 18 strip cropping farmers that had started implementing strip cropping in either 2019 or 2020 were identified. All farmers autonomously decided to start experimenting with strip cropping after having attended workshops and field excursions. With arable land in the Netherlands priced at around €100,000 per hectare, farmers experimenting at their own expense—even on one hectare—demonstrate a genuine commitment to strip cropping as part of their farming practices. We recruited the farmers based on availability, as well as the farmers’ representation in terms of diversity in soil types, production orientations, crops, strip cropping areas, and strip widths. Seven farmers (referred to as F1-F7), representing the mentioned diversity, confirmed their availability and were interviewed (Fig 1). Some farmers (i.e., F2, F4, and A9) applied experimental setup as they were co-innovating with researchers from universities and research institutes.

Two rounds of in-depth interviews were conducted. For the first round in April 2021, a semi-structured interview was designed and conducted following the guidelines by Emans [38] (S1 File). The interview guide consisted of four sections based on the requirements to understand the implementation of novel farming practices as proposed by Merot *et al.* [37]. The four sections were: (i) description of context and cropping systems in terms of crop species, temporal rotation, spatial allocation, and crop management; (ii) objectives and challenges in transitioning to strip cropping systems; (iii) outcomes of the first year of strip cropping implementation; and (iv) changes in crop management decisions caused by strip cropping. Sections (i), (ii), and (iii) in the interview guide address the first research objective and section (iv) addresses the second. In the second interview round in July 2021, results of the first round were discussed in terms of correctness. In most cases the farmers provided additional information to correct or extend the first-round results.

Two interviewers were present during each interview round. Interviews during the first round lasted between 1.5 and 3 hours and were held mostly online due to restrictions during the COVID-19 period. Interviews during the second round lasted for around 1 hour and were typically held at the farmer’s kitchen table. All interviewees had at least two years of farming experience. All interviews were conducted in Dutch except for one (F3) where English was used given the high English proficiency of the farmer. Conducting the interviews in the interviewee’s native language (Dutch) was considered to be the best approach to capture the authenticity and accuracy of the answers [39]. Written consent was obtained to record, transcribe, translate, and analyze the interviews. The transcripts were later translated to English before coding (see 2.2).

**Fig 2:**
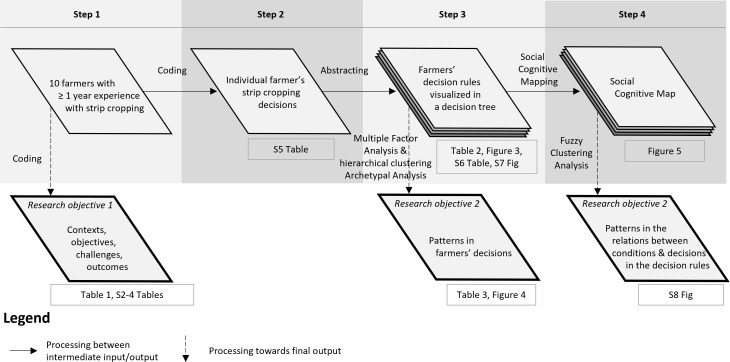
Diagram illustrating the flow of information and the results of each step of the approach used in this study. The final outputs which address the two research objectives are indicated in bold parallelograms. Boxes underneath the parallelograms indicate the figures and tables in Results and Supporting Information.

Data saturation was checked in three ways: 1) three additional farmer interviews (A8-A10; Fig 1); 2) comparison of results with a survey among 30 arable farmers who expressed interest to experiment with strip cropping systems on their perceptions on strip cropping; and 3) interviews with three agronomists that had been experimenting on-station for multiple years. Reaching data saturation would ensure that extra interviews would not yield new groups within the population of strip cropping frontrunner farmers in the Netherlands during the time of study.

### 2.2. Thematic coding

Two researchers independently coded each interview in Atlas.ti (Version 9.0.23.0). For each section in the interview guide—contexts, objectives and challenges, outcomes, and changes in decisions—we coded individual farmer statements deductively into themes and inductively by adding a new code if a new theme was addressed (step 1 in Fig 2). As excerpts on challenges and changes in decisions were often mentioned along with a specific crop management phase, we distinguished the different phases and used them to further organize these excerpts. Eight phases were distinguished: strategic planning, soil preparation, sowing/planting, fertilization, irrigation, spraying, weeding, and harvesting.

### 2.3. Abstracting farmers’ decision rules

Several decision models have been proposed to represent farmers’ resource management decisions. Merot *et al.* [37] proposed to represent farmers’ decision-making processes in the form of a ‘model of action’ [36], which links the status of a cropping system to the operational management decisions. Strip cropping decisions refer to operational management decisions by individual farmers that were considered specific for strip cropping (step 2 in Fig 2). A decision rule is an abstraction of a strip cropping decision of one or more farmers (step 3 in Fig 2), formulated as “IF <condition>, THEN <decision X>, ELSE <decision Y>, OR <decision Z>”. For example, IF machine width > strip width, THEN choose a wider strip width, ELSE rent/buy new machine, OR use the current machine as in sole-crop monoculture resulting in unintended effects on the neighboring strip.

We developed a decision tree to visualize the decision rules of the ten farmers (F1-A10), categorized by the eight crop management phases. Per phase, we allocated each decision rule in one or more of the four dimensions of diversity it mobilized (step 3 in Fig 2). The four dimensions of diversity are: genetic, temporal, and spatial, as proposed by Ditzler *et al.* [21], and operational crop management, as proposed by Carrillo-Reche *et al.* [40]. The eight management phases and four dimensions result in a total of 32 combinations, however not all combinations were encountered in the data.

We evaluated if the interviews with the three additional farmers (A8-A10) yielded new decision rules and compared the decision tree with the survey among thirty arable farmers across the Netherlands who expressed interest to take up strip cropping systems. No new decisions, objectives, or challenges were encountered from the comparison with the additional farmers and the survey, indicating that data saturation was reached. The interviews with agronomists did reveal new strip cropping decision rules that were then added to the tree.

### 2.4. Identifying patterns in farmers’ decision rules

To address the second research objective, we employed two analytical lenses to identify patterns in farmers’ decision rules following uptake of strip cropping at two abstraction levels. The first analytical lens, which comprised two sub-lenses, was employed to explore clusters and archetypes of farmers based on their decisions (step 3 in Fig 2). The second lens was employed at a higher abstraction level. We further abstracted the conditions and the decisions that constitute the decision rules, and clustered the farmers based on the relations between the abstracted conditions and decisions (step 4 in Fig 2). The results of these two lenses were compared to identify patterns in the farmers’ strip cropping decision rules.

#### 2.4.1. First lens: Multiple Factor Analysis followed by hierarchical clustering and Archetypal Analysis.

Multiple Factor Analysis (MFA), a generalization of Principal Component Analysis (PCA), is a common method in various domains including economy, ecology, and agriculture to analyze multiple sets of variables measured on the same objects [41]. Here, MFA was exploratively used to cluster individual farmers described by the presence or absence of strip cropping decisions, called functional variables in the terminology of MFA [41,42]. The absence of a decision means either that it was not considered by a farmer or that it was not considered relevant to address a given condition. Since MFA allows analysis of several sets of functional variables, we used different sets of decisions and evaluated which sets would capture most information in the data. MFA converged without overfitting warnings. Four sets emerged as the optimal sets. These were synthetic topics on objectives, spatio-temporal configuration design, crop neighbors, and strip width (see 3.2). Considering the balance between dimensionality reduction and information retention, we examined only the first two principal components. Together they explained 40.4% of the total variance, which was of similar value as used in Morel *et al.* [18]. Next, hierarchical clustering was conducted using Ward’s method [43]. The optimal number of clusters were determined using the elbow method by inspecting the residual sum of squares (RSS). We then proceeded with the Archetypal Analysis, which, like MFA, is suitable for quali-quantitative data analysis, to see if this procedure resulted in recognizable and comparable clusters.

Archetypal analysis (AA), first introduced by Cutler and Breiman [44], is an unsupervised learning method aimed to find ideal types, the archetypes, within a set of multivariate observations as convex combinations of extremal points—those exhibiting distinctive strategies of the frontrunner farmers rather than typical observations or cluster centers as in MFA [45,46]. Archetypes represent quintessential examples rather than actual entities, with the loadings quantifying the degree of membership association to each archetype [47,48]. A key advantage of this method is its ability to identify archetypes from small samples while capturing outlier cases often overlooked by conventional clustering methods [46,48]. AA has recently been used to identify and categorize response patterns in a specific context both at regional level, such as on land use governance and vulnerability to climate and global changes [49–52], and at household farm level on agroecological practices and climate change adaptation [46,48,53]. As with MFA, here AA was used to identify frontrunner farmer archetypes based on the presence or absence of their strip cropping decisions. The optimal number of archetypes was determined by evaluating the residual sum of squares (RSS) and the Akaike information criteria (AIC) where a balance between model complexity and the improvement in model fitting is struck. The farmers were then assigned to an archetype using the two-thirds criterion: no membership (loadings of 0–0.33), partial membership (0.33–0.67), and full membership (0.67–1.00) [cf. 46, 48, 53].

#### 2.4.2. Second lens: Social Cognitive Mapping followed by fuzzy clustering.

Cognitive maps graphically represent structures of interlinked elements in the form of directed graphs comprising nodes connected by directed edges that show causal relationships [Axelrod et al., 1976, as cited in 54]. While cognitive mapping approaches focus broadly on mapping causal relationships within a system without necessarily focusing on actors’ specific practices, the Cognitive Mapping Approach for analyzing Systems Of Practice (CMASOP) is developed to specifically gain a detailed understanding of how actors organize their practices [54,55]. Here we applied the CMASOP designed for understanding the complexity of farming systems management [54,56] to analyze the relations between the abstracted conditions and decisions that constitute the decision rules. A Social Cognitive Map (SCM) was developed by aggregating the individual cognitive maps of the ten farmers.

In the SCM the nodes represent either the abstracted conditions or decisions that constitute the strip cropping decisions rules. The edges represent unidirectional relations from a condition node to a decision node. A weight of 1 was assigned to an edge if it was mentioned or implied at least once by a farmer, otherwise 0 was assigned. The latter could arise either because the link between the nodes was not considered by a farmer or because the decision tied to a condition was not considered as a workable option. We analyzed the SCM using the graph theory indicator centrality, which is the cumulative weight of edges leaving a condition node or going into a decision node. A high centrality value of a node indicates that farmers attributed high importance to the node.

To identify (dis)similarities between the farmers, a dissimilarity matrix was computed using the Sorensen coefficient to perform fuzzy c-means clustering analysis on the edges of the SCM (step 4 in Fig 2). We used the Sorensen coefficient which is an asymmetrical similarity coefficient for the analysis of binary data (i.e., the presence/absence of edges between objects), whereby joint absence (in contrast to joint presence or a presence-absence combination) does not provide information on resemblance [56]. This applies to our case where the absence of an edge among two farmers does not provide relevant information for an indication of similarity between farmers. The quality of the clusters was evaluated using the normalized Dunn’s partition coefficient expressed on a scale from 0 (fuzzy clusters) to 1 (crisp clusters).

All analyses were conducted in R (Version 4.0.2) using the packages FactoMineR [57], factoextra [58], ade4 [56,59], archetypes [45], and RgraphViz [60].

## 3. Results

### 3.1. Farmers’ first year of strip cropping implementation

#### 3.1.1. Contexts.

Farms are located across the Netherlands on soils ranging from clay to sand textures. Farmers associated their farms with four production orientations: conventional, transitioning to organic, organic, and biodynamic. The area under strip cropping in 2020 varied from 1 to 64 ha; strip widths ranged from 1.2 to 22 m. The crop rotation length was either six or eight years involving a minimum of four arable crops. Cereals were present in all farms except F7 (Table 1).

**Table 1:** Context and cropping systems characteristics of the ten farmers interviewed in 2021 on their experiences with first-year strip cropping.

Farm code	Province	Soil type	Production orientation	Crops in the strip cropping system	Area under strip cropping in 2020 (ha)	Strip width (m)
F1	Zeeland	Clay	Biodynamic	Bean, chicory, cereals, grass-clover, potato, pumpkin	8	3
F2	Zeeland	Clay	Conventional^1^	Carrot, cereals, onion, sugar beet	4	6, 12
F3	Gelderland	Sandy-loam	Organic	Cauliflower, grass-clover, leek, lettuce, cereal-bean, pumpkin, spinach	1	1.5
F4	Flevoland	Clay	Organic	Bean, broccoli, celeriac, cereal, grass-clover, onion, parsnip, potato	64	6
F5	Groningen	Sand	Conventional	Cereals, hemp, maize, sugar beet	8	6
F6	Drenthe	Sand	Biodynamic	Bean, cereals, lupin, maize, pea	20	6
F7	Zuid-Holland	Clay	Organic	Cabbages, carrot/parsnip, chicory, grass-clover, onion, potato, pumpkin	7	3
A8	Zuid-Holland	Sandy-loam	Organic	Beans, beets, cabbages, carrot/parsnip, celeriac, cereal, leek, onion, potato, pumpkin, zucchini	1.7	1.2
A9	Flevoland	Clay	Conventional^1^	Bean, carrot, cereals, grass-clover, onion, potato	23	3, 22
A10	Groningen	Clay	Organic in transition	Cabbages, cereals (including traditional varieties), lucerne, pumpkin, sugar beet	± 4	12

^1^ Experimental farm conducting agricultural research in collaboration with universities and research institutes

#### 3.1.2. Objectives for adopting strip cropping.

From the 18 objectives mentioned by the ten interviewed farmers (S2 Table), five objectives were shared by more than 50% of interviewees. Increasing insect biodiversity was identified by all farmers as an important incentive to implement strip cropping. Improving soil quality and increasing natural enemies were objectives shared by seven and six farmers, respectively. The objective to experiment with “*what works?*” and “*what doesn’t work?*” and the objective to be an example for their region, were each mentioned by six farmers as reasons to implement strip cropping. For the latter, farmers mentioned that they would like to show other farmers what they do, what strip cropping can look like, which problems arise, and what results are obtained.

Aesthetic appeal was mentioned by three farmers (F1, F4, and F7). F4, located in a peri-urban area next to a highway, arranged their strip cropping fields perpendicular to the road. This not only highlights the aesthetics of strip cropping but also reconnects urban and rural communities, reducing polarization between them. The objective to increase or maintain yield and revenue compared to sole-crop monocultures was mentioned only by two farmers (F2 and A9). Another two farmers, F1 and F7, mentioned passing on the farm to the next generation and producing food sustainably as objectives for transitioning to strip cropping. Both considered strip cropping only as the first step towards a more diversified farming system needed for sustainable food production.

#### 3.1.3. Challenges in adopting strip cropping.

We identified 20 challenges in the first year of strip cropping implementation. Some challenges concerned all crop management phases, while others were linked to specific phases (S3 Table).

All farmers identified the lack of theoretical agro-ecological knowledge and practical experience in planning and implementation of mechanized strip cropping systems as a challenge that affected all crop management phases. As pointed out by F3, “*Strip cropping does not simply mean having strips of different crops close to each other. … the challenge is to find out what works for machinery, and also the combination of the crops.*” As crops in strip cropping systems are in close proximity to each other compared to a sole-crop monoculture, a higher degree of strategic planning is required to allow timely (independent) management of the different crops in neighboring strips. For example, F4 learnt and described that a delay in sowing green manure crops was necessary so that the strip later hosting the green manure crop could be driven over to harvest a neighboring crop.

Choice of working width was identified by eight farmers as a challenge linked to all crop management phases, especially sowing, irrigation, fertilization, spraying, and harvesting. In general farmers had already considered the feasibility of their chosen strip width during the development of their strip cropping plan. However, in practice, fully-mechanized operational management with current machines was challenging as it often involved adjusting machines to fit the chosen strip width or allow independent management of the different strips. When this was not possible, farmers bought new machines or rented from other actors. Similarly, damage to crop neighbors was a challenge shared by eight farmers with regards to irrigation, spraying, and weeding. For example, F3 and F6 mentioned that sometimes they irrigated neighboring crops they would rather not irrigate. Not only was this considered inefficient, but also risky as it could lead to fungal diseases that would damage the crops especially in the early growing stages.

Some challenges were mentioned by a few farmers only. For instance, crop choice in relation to inflexibility to react to yearly market changes was mentioned by one farmer (F2). Similarly, communication with contractors, to whom operations such as sowing or harvesting are outsourced, was mentioned as a challenge by three farmers (F6, F7, A10) due to the lack of experience of the contract workers in working with strip cropping systems. For example, F6 instructed the contract workers to unload the grain harvest in the combine at the end of each strip to prevent unnecessary soil compaction by driving a tipper on the neighboring strip. However, they disregarded the instruction and stuck to the common practice in monocultural system of a combine harvester accompanied by a tipper.

#### 3.1.4. Outcomes of the strip cropping implementation.

Farmers mentioned various outcomes from their first-year strip cropping experience. Outcomes were qualitative (i.e., positive, neutral, or negative) as farmers generally did not collect quantitative data to support their observations. We summarized the outcomes in 22 indicators (S4 Table).

While farmers were not (yet) able to find workable solutions to all the challenges (S3 Table), most farmers associated their first-year strip cropping experience with positive outcomes. Farmers unanimously mentioned a positive outcome on the availability and feasibility of mechanization for strip cropping implementation. They had found workable solutions by using, adjusting, renting, or buying (new) machines. Increased workload was mentioned as negative outcome by most farmers (n = 8). Seven farmers mentioned a neutral outcome on yield in strip compared to sole-crop monoculture. If a farmer mentioned a negative effect on yield, it was often associated with non-optimal weather conditions rather than strip cropping. Two indicators had contrasting evaluations among farmers: pest and disease incidences and size of natural enemy population. While effect on pest and disease incidences was positively evaluated by five farmers, it was negatively evaluated by two. Similarly, the size of natural enemy population had two positive and one negative evaluations.

### 3.2. Farmers’ decision rules

Individual farmer’s decisions in response to the uptake of strip cropping were recorded per crop management phase and crop (S5 Table). From these, 49 decision rules comprising 11 conditions and 43 decisions were developed (S6 Table). The conditions identified here were always tied to specific decisions and represent only a subset of the objectives and challenges identified in sections 3.1.2 and 3.1.3. Table 2 illustrates part of the list of the decision rules on crop neighbor during the harvesting phase.

**Table 2:** Decision rules summarizing farmers’ decisions related to crop neighbor conditions during the harvesting phase. Responses indicate the number of farmers that mentioned each strip cropping decision rule. The column describing the code in the decision tree shows how the rules are represented in the decision tree (see S7 Fig).

Crop management phase	Decision rule	Example of relevant crops	Responses	Code in decision tree
Harvesting	*IF* strip width equals harvester width *AND* neighboring strip is not required to unload and store the harvest, *THEN* keep strip cropping plan	Leek, cereals, bean, potato, onion, alfalfa	6	N_avail4
	*ELSE*			
	*IF* current (own) machine modification is possible *AND/OR* financial means are sufficient, *THEN* either adjust current harvester or buy a new machine to harvest and load within the strip, e.g., a bunker harvester with sufficient storage volume	Sugar beet, cabbage, pumpkin, potato, chicory, onion, carrot harvested by potato harvester	4	N_avail2
	*ELSE*			
	*IF* possible to rent from other actors, *THEN* rent from other actors to harvest and load within the strip with, e.g., bunker harvester	Sugar beet, potato	2	N_avail3
	*ELSE*			
Strategic planning, harvesting	sow a neighboring crop that either can be driven over (e.g., green manure, grass–clover) or a crop with earlier harvest date	Parsnip or carrot with grass–clover as neighbor; broccoli, celeriac, or potato with a mixture of oats, clover, vetch and phacelia as neighbor; sugar beet with a cereal as neighbor	3	N_avail1
Harvesting	*IF* a crop grows within the strip boundary, *THEN* use current (own) machine as in sole-crop monoculture	Any crop	0	N_bound4
	*ELSE*			
	*IF* there are varieties that grow upright, *THEN* use upright varieties (e.g., pumpkin variety Butter nut instead of Orange summer or Big banana)	Pumpkin	2	N_bound1
	*ELSE*			
	*IF* current (own) machine modification is possible, *THEN* adjust current (own) machine to trim the vines growing outside the strip boundary	Pumpkin	1	N_bound2
	*ELSE*			
	hand-harvest produce that are growing outside strip boundary	Pumpkin	1	N_bound3

**Fig 3:**
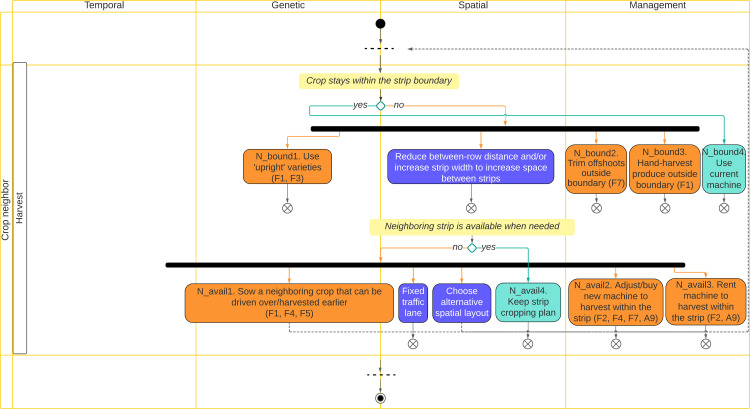
Illustration of part of the decision tree built to visualize the synthesized farmers’ decision rules based on the part of the list of the decision rules presented in Table 2. Columns in the diagram represent the four dimensions of diversity: time, genes, space, and operational crop management. The rotated text on the left side of the tree shows one of the four synthetic topics for one of the eight management phases. Here results are shown for the topic Crop neighbor and management phase Harvesting. Farmers’ decision rules and farmer codes are presented in green and orange boxes. Blue boxes represent decision rules proposed by agronomists. Dotted arrows represent feedback loops.

The decision rules were visualized in a decision tree (Fig 3, S7 Fig). We used the four sets of functional variables in the MFA (see 2.4.1) to divide the tree and arrange the decision rules into four synthetic topics: Objectives, Spatio-temporal configuration design, Crop neighbors, and Strip width. The Objectives topic covered the decision rules that started with conditions on strip cropping objectives to improve biodiversity, pest and disease control, and soil quality. The other three topics covered decision rules that started from conditions on: design of spatio-temporal configuration of strip cropping system, interactions among neighboring crops, and strip width in relation to current machine width, respectively.

Consultation of agronomists yielded 24 new decision rules, for example combining perennial, summer, and winter crops in the rotation or using competitive species to address weed pressure (see blue boxes in Fig 3 and S7 Fig). Almost half of the new decision rules mentioned by the agronomists involved decisions already mentioned by the farmers for other conditions. For example, adjusting between-row distance and/or between-strip distance was mentioned by F7 to address crop neighbor damage, while according to the agronomists it could also be used to address crops growing outside the strip boundary and address weed pressure by allowing mechanical hoeing or reducing between-row distance to close crop canopy.

The operational crop management dimension was mobilized in more than half of the farmers’ decision rules, while genetic, spatial, and temporal dimensions were mobilized less (18%, 14%, and 12%, respectively). Combining both farmers’ and agronomists’ decisions, most still operated in the operational crop management dimension (40%), followed by spatial, genetic, and temporal (26%, 21%, and 14%, respectively).

### 3.3. Patterns in farmers’ decision rules

#### 3.3.1. First lens: Multiple factor analysis followed by hierarchical clustering and archetypal analysis.

**Fig 4:**
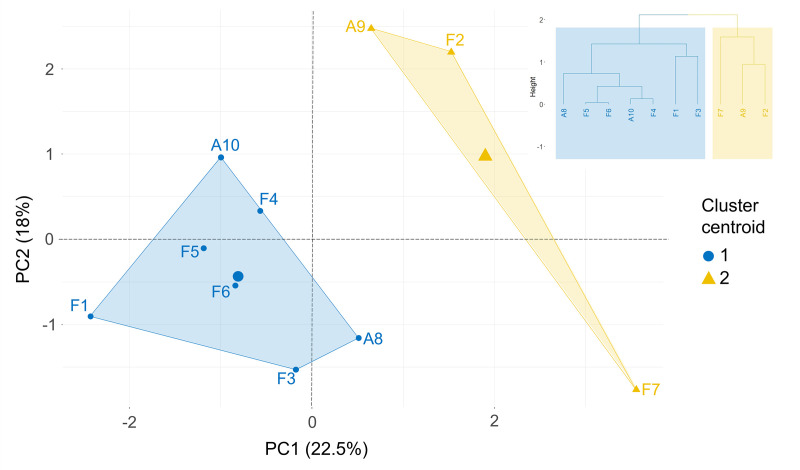
First two principal components of the factor map of the hierarchical clustering of farmers (F1-7 and A8-10) based on the MFA. The farmer codes are presented in Table 1. The formation of the two hierarchical clusters in a dendrogram is presented at the top right corner of the factor map.

Comparison of both analyses converged in revealing that the two clusters from MFA followed by hierarchical clustering contained the same farmers as the two archetypes from AA (Table 3, Fig 4). From the MFA, the clusters which together explained 24.8% of the variance, were projected onto the first two principal components (Fig 4). The first principal component was defined by Crop neighbor (35.9%) followed by Objectives (24.8%), while the second principal component was defined by Strip width (33.0%) and Crop neighbor (32.7%). In the AA, upon applying the threshold of two-third criterion, all 10 farmers mapped fully onto one of the two archetypes (Table 3).

**Table 3:** Loading of the ten farmers onto the two archetypes. Shading and shade color (reflecting MFA cluster colors) represent full archetype membership (loadings ≥ 0.67).

Farm code	Loading onto Archetype 1	Loading onto Archetype 2
F1	0.999	0
F2	0	0.999
F3	0.966	0.033
F4	0.856	0.143
F5	0.999	0
F6	0.999	0
F7	0	0.999
A8	0.800	0.198
A9	0.095	0.904
A10	0.888	0.111

Cluster and Archetype 1 were both composed of F1, F3, F4, F5, F6, A8, and A10, who shared two decisions that set them apart from the other. The first was to keep the strip cropping plan (N_avail4 in S3 Table) when the neighboring strip is available for harvesting the crop next to it. Secondly, all farmers who did not mention or consider adjusting or buying new machine to harvest within the strip (N_avail2) when the neighboring strip is unavailable during harvest were assigned to this cluster. As such, this cluster and archetype could represent farmers who were less machine-oriented in fitting strip cropping into their current farming system.

In contrast, Cluster and Archetype 2 were both composed of F2, F7, and A9, who were more machine-oriented while considering other (agroecological) options in fitting their (envisioned) farming systems and machinery into the conditions of strip cropping. For example, they mentioned renting, adjusting, or buying new machines to harvest within the strip when the neighboring strip is unavailable (N_avail2, N_avail 3) and when machine width is larger than the strip width (W_wa5 and W_wa6). To address the latter during spraying, irrigating or fertilizing, they also considered using section closure (W_wa4). While F2 and F7 mentioned postponing spraying to explore the potential of biocontrol (O_bb1) and using green cover to lower compaction while driving (O_soil1), F7 and A9 mentioned using fixed traffic lines to address the objective of reducing soil compaction (O_soil2).

#### 3.3.2. Second lens: Social cognitive mapping and fuzzy clustering.

Using the second analytical lens, farmers were clustered based on the relations between the abstracted conditions and decisions that constituted the decision rules. Fuzzy clustering did not result in identification of farmer clusters, as indicated by a normalized Dunn’s partition coefficient of 0.07 (S8 Fig). This result implies that no clear pattern on the relations between conditions and decisions in the decision rules could be derived.

**Fig 5:**
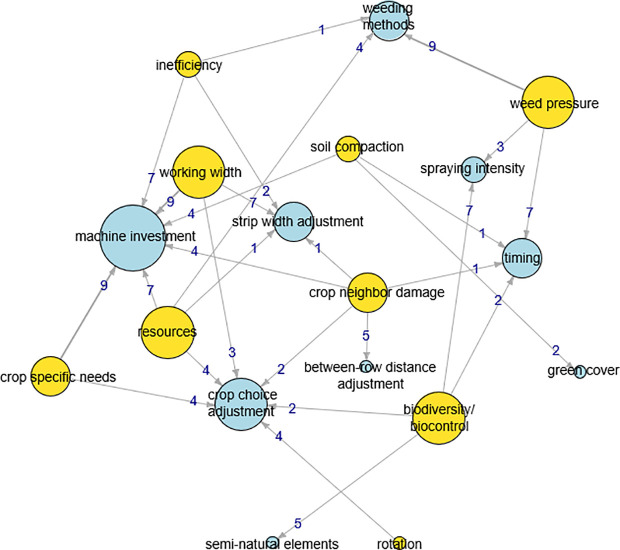
Social Cognitive Map (SCM) of the ten interviewed farmers with nine condition nodes (yellow) and nine decision nodes (blue). Table 4 provides the explanation of the nodes. Node size corresponds to the centrality (i.e., sum of edges leaving a condition node or entering a decision node) and was scaled into five sizes for visualization purpose: the smallest circles represent a centrality of 1-5, followed by centrality values ranging between 6-10, 11-20, 21-30, and the largest circle with centrality greater than 30. Edge line thickness corresponds to the number of times the edge was encountered in the data.

In total, 18 nodes (nine condition nodes and nine decision nodes) connected by 113 edges were identified (Table 4, S6 Table) and visualized in a Social Cognitive Map (SCM) (Fig 5). No edge between condition and decision was shared by all farmers. Nine farmers mentioned the relation from: “weed pressure” to “weeding methods”; “working width” to “machine investment”; and “crops specific needs” to “machine investment”. In contrast, the relation from: “inefficiency” to “weeding methods”; “soil compaction” to “timing”; “resources” to “strip width adjustment”; and “crop neighbor damage” to “strip width adjustment” and “timing” were mentioned by one farmer.

**Table 4:** Explanation of condition nodes and decision nodes in the SCM, ordered by decreasing centrality.

Condition node	Decision node	Explanation	Centrality
	Machine investment	Use current machine, adjust, rent, or buy machines	40
Weed pressure		Current weed pressure and expected build-up	19
	Crop choice adjustment	Adjust the crop choice, think in terms of and utilizing crop families, species, and/or cultivars	19
Working width		Machine width in relation to strip width (i.e., equal width, wider, or narrower)	19
Resources		Resources for investments	16
Biodiversity/ biocontrol		Improving biodiversity and/or biocontrol is stated as an objective	16
	Weeding methods	Use different weeding methods, e.g., false seedbed, flame, hand, mechanical, spraying	14
Crop specific needs		Crop-specific needs during the various crop management phases	13
Crop neighbor damage		Damage on neighboring crop due to management operation (e.g., mechanical, over-irrigation, spraying damage)	13
	Timing	Change the operation timing and/or frequency of different crop management phases	11
	Strip width adjustment	Change strip width	11
Inefficiency		Potential inefficiency resulting from particular management	10
	Spraying intensity	Reduce the intensity of spraying (including not spraying)	10
Soil compaction		Reducing soil compaction is stated as an objective	7
	Between-row distance adjustment	Adjust the between-row distance within a strip	5
	Semi-natural elements	Use semi-natural habitat on- or around the field	5
Rotation		Problematic rotation due to, e.g., carry-over effects of pest and diseases	4
	Green cover	Use (year-round) green cover or break crop to cover the soil	2

The four condition nodes with greatest centrality, ranging between 16 and 19 were “weed pressure”, “working width”, “resources”, and “biodiversity/biocontrol”. These conditions were mentioned in relation to three or four decisions. Although having a centrality of 13, “crop neighbor damage” stood out as a condition node that was linked to five decision nodes. This implies that “crop neighbor damage” affected more farmers’ decisions than the other conditions. “Machine investment” (centrality 40) and “crop choice adjustment” (centrality 19) emerged as the two most important decision nodes as they were both often mentioned by the farmers and each linked to six condition nodes. Together, these decision nodes were linked to all condition nodes except “weed pressure”.

## 4. Discussion

### 4.1. Summary of results

We studied how ten frontrunner farmers experienced their first year of transitioning from sole-crop monoculture to strip cropping systems. The objectives shared by most farmers were increasing insect biodiversity, enhancing soil quality, experimenting with strip cropping, and being an example for other farmers in the region. The lack of agro-ecological knowledge and practical experience, incompatible working width, and crop neighbor damage were the most common challenges in implementing fully-mechanized strip cropping. The majority of farmers positively evaluated the feasibility to adjust or acquire adapted machines for strip cropping, were neutral on changes in yield, and negatively evaluated workload. Based on the farmers’ accounts of their management, we formulated 49 decision rules comprising of 113 relations that connected nine condition nodes to nine decision nodes. In identifying patterns in farmers’ decision rules in transitioning from sole-crop monoculture to strip cropping, from the first analytical lens, we observed two clusters or archetypes of farmers who differed in their propensity to adjust mechanization. From the second lens, we did not find distinct patterns in the relation between condition and decision in farmers’ decision rules. Nonetheless, we found “crop neighbor” as a key condition and “machine investment” and “crop choice adjustment” as the two most mentioned decision nodes.

### 4.2. Objectives and challenges in diversified systems

The objectives and challenges identified in this study agree with those found in broader studies on adoption of crop diversification strategies, including intercropping. For example, similar as in this study, improving soil quality and biodiversity was identified to be among the main objectives across 80 intercropping farmers in Europe [61]. Higher yield was considered less important by farmers [61], although maintaining or increasing yield is often mentioned as one of the main objectives in agronomic research on intercropping systems [62–64]. In our sample, seven farmers mentioned yield as one of the indicators, among which, two experimental farm managers also mentioned it as an objective. Financing new technology and the lack of technical knowledge were among previously identified challenges to crop diversification practices in Europe [18,65,66]. Damage in neighboring crops and crop specific needs for irrigation, fertilization, weeding, and spraying were newly identified challenges in this study.

### 4.3. Absence of distinct patterns based on the two analytical lenses

Clustering farmers allows for identifying and understanding patterns in the diversity of farmers’ adaptation strategies [67], in this case, following strip cropping uptake. The first lens identified two clusters or archetypes of farmers based on their operational decisions. These clusters or archetypes reflect two tendencies: one that integrates strip cropping into their existing farming systems with a lower emphasis on machine-orientation, and the other that adopts a more machine-oriented approach, incorporating additional (agroecological) practices such as green cover for soil compaction and biocontrol measures to enhance biodiversity. However, these clusters or archetypes are associated with few decision, tied to farmers’ context, e.g., access to alternative mechanization. At a higher abstraction level from the second analytical lens, the poor quality of fuzzy clustering and the limited number of edges shared by nine farmers in the SCM pointed to the absence of distinct pattern in farmers’ decision rules.

### 4.4. Factors that might explain dissimilarities among frontrunners

Farmers’ decision-making is influenced by many factors, among which are farm context and management strategy which is influenced by the values, preferences, and formal educational background of the farm manager [30,68–70]. From the first lens, we observed that decisions on tackling pumpkin vines that grew outside the strip boundary were only relevant in the context where pumpkin was grown in strips (F1, F3, F7, A8, and A10; Table 1). However, only F1, F3, and F7 mentioned decisions on that. The other two farms either did not find it challenging, had not found a solution, or simply forgot to mention it. In the SCM the condition on working width linked to the decision on machine investment was irrelevant for A8 given that most crop management was done manually by the farm’s consumer community members.

Given the early stage of development surrounding strip cropping in the Netherlands, how the frontrunner farmers adapted their farming practices may not have stabilized yet. Farmers may still be undergoing “step-by-step design” processes [71]—constantly assessing, generating creative solutions, and tailoring their solutions to their contexts [72]—partially due to the lack of contextualized references from research [73]. While there is an emerging body of empirical underpinning for the ecosystem services provided by strip cropping [see, e.g., 19, 74–76], practical implementation varied as it is still in the trial-and-error realm. This can explain the observation that the frontrunners were creating their own “fingerprint” of diversification strategies [77,78]. For example, in the SCM, crop neighbor damage was a condition linked to five decisions, with each link mostly mentioned by one to two farmers. This indicates variation in how different farmers took different decisions in response to the same condition. Even when there was a clear consensus on the link between condition and decision, for instance as seen from the nine edges between weed pressure and weeding, the decisions on handling the weeds differed among farmers. These decisions ranged from using mulch, pre-crop to clean the soil, mechanical/ manual/ chemical weeding, to not weeding. Similarly, given the same condition on pumpkin vines growing beyond the strip boundary, F1 and F3 decided to use ‘upright’ varieties while F7, given his high degree of technological affinity and adeptness, chose to modify the machine by adding cutting blades on both sides of the harvester and conveyer belt to allow harvesting within the strip.

Adoption of a new farming system is a dynamic process that evolves over time. It often takes several years for farmers to familiarize themselves with the new system and build experiences [79,80]. For example, Revoyron *et al.* [78] found clusters of farmers who differed based on their motivation, speed of evolution, and extent of crop diversity when analyzing diversification pathways of 33 farmers with 2–26 years of experience. The result of this study was obtained with a small absolute sample size, even if constituting half of the strip cropping frontrunners and covering most, if not all their decision rules in the Netherlands at the time of the study. As the population of innovators grows and as experience mounts in the later stages of the transition-in-the making, distinct patterns in farmers decision rules following uptake of strip cropping may be revealed. Clusters are dynamic in nature, which might result in farmer reassignment into a different cluster as their expertise, focus, and thus practices evolve over time. For example, A10, who was mapped in Cluster 1 in the first analytical lens, may now align with Cluster 2 due to his increased technological adeptness, as demonstrated by under sowing cabbage using drones. Thus, further studies would be warranted to reveal the variability in farmers’ decision-making processes over time in relation to different farm contexts, both more specifically in the Dutch context (e.g., the ongoing research in the CropMix project (www.cropmix.nl/en)) and in the broader European contexts. This includes longer-term longitudinal studies to track the evolution of strip-cropping adoption and comparative analyses to examine how these processes change over time and with experience. Altogether these insights can inform interventions tailored to enable broader implementation of strip cropping in diverse agricultural settings [81,82].

### 4.5. Usefulness of the approach

Tapping into the flow of unformalized experience of frontrunners, we aimed to assist aspiring and current strip cropping farmers, advisors, and researchers in the design and development of farm-specific strip cropping systems. Feedback to the interviewees and presentations to aspiring farmers during strip cropping training courses on the decision tree and the SCM prompted three types of effects. First, presenting the diverse decisions of the different farmers, instead of only the ‘star’ farmers (e.g., F4, F7, and A9), stimulated reflection and exchanges. This may be considered learning on the changes in decisions due to uptake of strip cropping by sharing perspectives and co-creation of knowledge, referred to as social learning [83]. Second, the SCM allowed to focus exchanges on the key condition “crop neighbor damage” that affected more farmers’ decisions than the other conditions and on the key decisions “machine investment” and “crop choice adjustment” that related to almost all conditions. These key condition and decisions may change in the future when the farmer population changes in composition. For instance the proportion of organic farms in the current sample of frontrunner farmers (50%) was high compared to the national average of 3.5% organic farms in 2020 in the Netherlands [84]. Lastly, the results provided space for discussing current lock-ins in farmers’ thinking. For example, none of the interviewed farmers considered using competitive crop species to address weed pressure (cf. SCM in Fig 5), while scientific evidence exists that such solutions are available (cf. decision tree in S7 Fig).

Fostering integration between formal and informal knowledge is necessary to enhance innovation processes that enable a wider uptake of more sustainable farming practices [85–88]. The documentation and sharing of informal knowledge paints realistic expectations [89] in terms of challenges, changes in decisions, and outcomes of the first year of strip cropping implementation. This could inspire other farmers, including those who are skeptical of the practice, thereby accelerating a wider strip cropping implementation.

The various links between conditions and decisions that we found farmers to make in their decision rules, could be thought of as multiple innovation pathways tailored to each specific local context. Transition studies have shown that transitions would benefit from pluralizing pathways as these provide multiple entry points for a wider context application by different contexts of farmers [90–92]. While currently there seem to be two entry points in terms of machine-orientation in fitting strip cropping to the current system or the other way around, as represented by the two farmer clusters or archetypes, more diverse entry points may emerge over time with experience. This would in turn facilitate enabling environment for further experimentation, advancing development of ‘out of the box’ innovations needed for disruptive changes [91,92]. The non-decisive pattern and the diversity in frontrunner farmers’ decision rules implies that a prescriptive approach is not productive to advance the evolution of strip cropping technology. Instead, capturing and keeping track of this diversity would facilitate entries of aspiring farmers from various contexts by allowing them to choose and prioritize which conditions to focus on and accordingly decide on the (extent of) changes they are willing to make their decisions upon [79,93].

Finally, the common challenges of the lack of agroecological knowledge, available technology, and market access identified in this study highlight the need for capacity building, along with infrastructure and institutional support beyond farmers’ control to enable broader strip cropping implementation. First, transdisciplinary research can build together agroecological knowledge with farmers, particularly regarding crop combinations and their interactions, assisting farmers in finding guidelines for good crop neighbors for their cropping system [40]. The transdisciplinary research should also include exploration of a wider range of indicators that would enable system-level evaluation of the strip cropping system that farmers base their decisions upon. These include yield stability and economic profitability that capture performance at the cropping system level rather than at the crop level and (additional) operational cost, which are valuable indicators for farmers but currently under-researched [25,26,61,94].

Infrastructure and institutional support, such as development of small-scale affordable autonomous machines designed for strip cropping, could improve efficiency and address issues related to workload, working width, and crop neighbor damage. Subsidies might be needed for compensating initial costs of adopting strip cropping [95]. To address the challenge of field registration, authorities could allow fields to have multiple crops, replacing the current system where each strip must be registered as an individual field [96,97]. Subsequently, developing (short) supply chains that can accommodate the diversity of the resulting produce would help address market access challenges. Lastly, a deeper understanding of farmers’ diverse decision rules in strip cropping could help policymakers, advisors, and researchers develop tailored interventions to scale context-specific strip cropping systems.

## 5. Conclusion

Strip cropping is an example of hybrid innovation niches that can facilitate transitioning towards more sustainable agricultural systems for actors outside and within the dominant agricultural regime. We tapped into the experience of ten frontrunner farmers and presented their contexts, objectives, challenges, implementation outcomes, and changes in their decisions following strip cropping adoption. We presented two clusters or archetypes of farmers with different propensity to adjust mechanization upon uptake of strip cropping. The lack of distinct pattern among the other adjustment from monocropping to strip cropping, along with the poor quality of clustering in the second lens indicated that changes were highly context- and farmer-specific. Nevertheless, the SCM analysis allowed us to highlight one key condition on crop neighbor damage that affected farmers’ decision more than the other conditions, and two prevailing decisions that were related to almost all conditions: investing in machine and adjusting crop choice. These conditions and decisions can serve as focal points for discussions, for example in strip cropping training events and farmer-to-farmer exchanges. Capturing the diversity in frontrunner farmers’ decision rules would stimulate social learning among farmers, their advisors, and researchers. Mobilizing this diversity through transdisciplinary research, along with tailoring infrastructure and institutional support to the specific needs of strip cropping, would facilitate a wider adoption of strip cropping, as a means towards more sustainable agricultural systems.

## Supporting information

S1 File Interview manual. (DOCX)

S2 Table Main objectives mentioned by the ten farmers for implementing strip cropping. (DOCX)

S3 Table Main challenges mentioned by the ten farmers in transitioning to strip cropping systems for the first time. (DOCX)

S4 Table Farmers’ evaluation of the outcomes of their first year experimenting with strip cropping. (DOCX)

S5 Table An example of the changes in operational management decisions that farmer F1 made when implementing strip cropping systems compared to sole-crop monoculture. (DOCX)

S6 Table List of 49 decision rules comprising 11 conditions and 43 decisions formulated based on the coded interviews with the farmers. (DOCX)

S7 Fig Decision tree visualizing farmers’ decision rules. (DOCX)

S8 Fig Cluster plot from fuzzy clustering of the ten interviewed farmers. (DOCX)
