# Supplementary material for: From sole crops to strip cropping: Decision rules of frontrunner farmers in The Netherlands
Source: PLoS One. 2025 Jul 24;20(7):e0329133. doi: 10.1371/journal.pone.0329133 (PMC12289020; doi:10.1371/journal.pone.0329133)
Supplement: S1 File — (DOCX) [file pone.0329133.s001.docx]

**S1 File: Interview manual**

**From sole crops to strip cropping: decision rules of frontrunner farmers in the Netherlands**

Stella D. Juventia ^1*^, Dirk F. van Apeldoorn ^1,2,3^, Hilde Faber ^1,3,4^, Walter A. H. Rossing ^1^

^1^ Farming Systems Ecology Group, Wageningen University & Research, Wageningen, the Netherlands

^2^ Field Crops, Wageningen University & Research, Edelhertweg 10, Lelystad, the Netherlands

^3^ Centre for Crop Systems Analysis, Wageningen University & Research, Wageningen, the Netherlands

^4^ Land & Co, Costerweg, Wageningen, the Netherlands

**S1 File. Guideline for the semi-structured in-depth interview.** The original guideline was written in Dutch, as our respondents are Dutch farmers. For interviewing F3 and for the purpose of this paper, we have loosely translated it to English.

**Interview manual**

**Information about the interview**

- Time and date of the interview:
- Attendees:
- Location of the interview:
- Language of the interview:
- Sex of interviewer & interviewee:
- Name of identification code of interviewer:
- Name interviewee:
- Case number:
- Recording (in case of online interview): Yes/No
- Use of the interview for scientific publication (highlight): Name farm, name farmer, anonymized

**The interview**

- Thank you for taking the time to answer our questions!
- A brief introduction of the interviewer(s) and the projects: TKI Agri & Food Public-Private Partnership (Transition to ecological circular agriculture through the application of crop diversity) and POP-3 Project (Cooperation Innovation Zeeland).
- Our goal with this interview is: to explore the decisions you make as a grower. We are talking about the first year after you have switched from regular monoculture to strip cropping. However, it is important that you tell your own story in this interview, regardless of what our purpose is with this interview. We chose you because we admire your work and think that your experience can offer us insights and inspiration. Especially because you are among the pioneers in strip cropping in the Netherlands.
- We first ask about the story of your company from just before and after the introduction of strip cultivation. We ask about the decisions you have made and the changes that have taken place during the transition to strip cropping. We would like to deal with these changes and decisions per crop, so that we can treat the crops in detail. This allows everything you find relevant to be discussed. Next, we may come back to certain points and ask for a few things to be worked out further.
- Before we begin, I'd like to ask permission to record this interview. The recording is not distributed and is only used for processing the information obtained. In addition, I would like to ask how you prefer to be named in the publications: Name farm, name farmer, anonymized?
- Let's get started!

1. **introduction**

1.1. Can you introduce yourself and your function at the farm? What are your company long-term ambitions or goals?

a. Yield, b. Soil/mechanization, c. Disease/pests (never really was a problem here), d. natural pest control, e. robust cultivation (resilience to extreme weather conditions), f. Biodiversity, g. Society, h. Fertilizer (new project), i. Others

1.2. How does strip cropping fit within those ambitions or goals? What is the role of strip cropping?

1.3. Can you tell us about what your business looks like now compared to before strip cropping?

1.4. How did you experience the transition to strip cropping?

1. **Timeline by crop**

2.1. What were the crops you grew last year and what was the crop rotation?

2.2. Now we would like to discuss the change and decisions you have had to make in the transition to strip cropping. To keep it clear, we would like to discuss this per crop, so one by one. For the same reason, we want to discuss the changes chronologically throughout the year. Per crop, from sowing/planting through the season to harvesting and planting any green fertilizers. Let's start with: ....; tell us how you have grown this crop in strips in the past year? How did this differ from how you managed sole-crop monoculture?

1. **Follow-up questions**

3.1. Are there any changes you had to make when you switched to strip cropping that have not yet been discussed?

a. Machinery, b. sowing dates, c. irrigation, d. ploughing, e. fertilization, f. spraying, g. Weed control, i. Crop combination

3.2. For *crop management phases in which a change has taken place*:

3.2.1. What did you want to achieve by applying this [action/change/strategy]?

3.2.2. Why did you choose this [action/change/strategy] over other possible solutions? Would you do things differently, if so how?

3.2.3. What were the results? Can you see changes in ....?

3.2.4. How did you know it was a success? (indicators) Can you give an example?
a. Yield, b. Soil/mechanization, c. Disease/pests, d. Natural pest control, e. Robust cultivation (resilience to extreme weather conditions), f. Biodiversity, g. Society (e.g. show other farmers, educate consumers etc.), h. Others

3.2.5. Why is this [indicator] important to you?

3.3. Where do you (envision to) sell your products? Do you think you will have any trouble with this? How would you deal with these difficulties?

3.4 What is your connection to the local society? How did it experience the transition to strip cropping?

1. **Lessons learned**

4.1. Just to check if 3.2.2 is complete: Looking back, would you make the same decision to achieve your goals? How else do you want to do it next year?

4.2. Finally, what tips or advices would you give to farmers who want to do strip cropping in the future?

1. **Closing**

5.1. Thank you for answering our questions, this was all on our part. Do you have any questions for us?

5.2 a. We can come back to you with some results so you can also learn from the experience of other 9 farmers who are also in their first three years of transitioning to strip cropping.

5.2 b. (optional) We would like to publish your story. Do you think it's okay that we write an article for *Ekoland* (magazine targeting organic Dutch farmers) about your farm story? You can check for inaccuracies or supplement them before we publish it.

5.3. You can always email for questions or if you would like to add something to your story afterwards or explain something even further. Hopefully we can do the same!
