# Supplementary material for: From sole crops to strip cropping: Decision rules of frontrunner farmers in The Netherlands
Source: PLoS One. 2025 Jul 24;20(7):e0329133. doi: 10.1371/journal.pone.0329133 (PMC12289020; doi:10.1371/journal.pone.0329133)
Supplement: S2 Table — (DOCX) [file pone.0329133.s002.docx]

**S2 Table: Main objectives mentioned by the ten farmers for implementing strip cropping**

**From sole crops to strip cropping: decision rules of frontrunner farmers in the Netherlands**

Stella D. Juventia ^1*^, Dirk F. van Apeldoorn ^1,2,3^, Hilde Faber ^1,3,4^, Walter A. H. Rossing ^1^

^1^ Farming Systems Ecology Group, Wageningen University & Research, Wageningen, the Netherlands

^2^ Field Crops, Wageningen University & Research, Edelhertweg 10, Lelystad, the Netherlands

^3^ Centre for Crop Systems Analysis, Wageningen University & Research, Wageningen, the Netherlands

^4^ Land & Co, Costerweg, Wageningen, the Netherlands

**S2 Table. Main objectives mentioned by farmers (n = 10) for implementing strip cropping.** Objectives are ordered in descending order of number of farmers’ responses. The objectives in bold were related to the strip cropping decisions that constituted farmers’ decision rules. The “O_” in the abbreviations refer to “Objective”; the abbreviations are used in the MFA analysis (section 2.3.2) and for the visualization of decision rules (S6 Table and S7 Fig).

| **Objectives (n = number of responses)** | **Farmers’ strip cropping decisions (abbreviation)** |
| --- | --- |
| **Increase biodiversity, specifically:** | - Add semi-natural habitat on/around field (O_bb2) - Consider crop species choice (O_bb3) - Do not spray and consider other options (O_bb5) |
| **• insect populations (n = 10)** |  |
| **• field bird populations (n = 3)** |  |
| **• bee populations (n = 1)** |  |
| **Improve pest & disease control through** |  |
| **• input minimization (n = 5)** | - Postpone spraying to explore the potential of biocontrol (O_bb1) - Add semi-natural habitat on/around field (O_bb2) - Consider crop species choice (O_bb3) - Use resistant/mixed varieties for biocontrol (O_bb4) - Do not spray and consider other options (O_bb5) |
| **• increased natural enemies population (n = 6)** | - Postpone spraying to explore the potential of biocontrol (O_bb1) - Add semi-natural habitat on/around field (O_bb2) - Consider crop species choice (O_bb3) - Do not spray and consider other options (O_bb5) |
| **• reduction of pest & disease pressure (n = 5)** | - Add semi-natural habitat on/around field (O_bb2) - Consider crop species choice (O_bb3) - Use resistant/mixed varieties for biocontrol (O_bb4) |
| **Improve soil quality (n = 7)** | - Use green cover to reduce compaction while driving (O_soil1) - Use fixed traffic lanes (O_soil2) - Use lighter machines (O_soil3) - Drive less frequently (O_soil4) - Distribute wheel pressure by e.g. operating the harvester side-by-side with a trailer to unload the harvest on neighboring strip (O_soil5) |
| Experimentation (n = 6) |  |
| Be an example for the region (n = 6) |  |
| Re-connect agriculture and nature (n = 5) |  |
| Fulfil responsibility to produce food sustainably  (n = 3) |  |
| Farming system diversification (n = 3) |  |
| Aesthetic appeal (n = 3) |  |
| Spread risk of crop loss (n = 2) |  |
| Pass farm on to next generation (n = 2) |  |
| Stabilize income through increased/ maintained yield (n = 2) |  |
| Farm income diversification (n = 2) |  |
| Maintain heritage (n = 1) |  |
