# Supplementary material for: From sole crops to strip cropping: Decision rules of frontrunner farmers in The Netherlands
Source: PLoS One. 2025 Jul 24;20(7):e0329133. doi: 10.1371/journal.pone.0329133 (PMC12289020; doi:10.1371/journal.pone.0329133)
Supplement: S3 Table — (DOCX) [file pone.0329133.s003.docx]

**S3 Table: Main challenges mentioned by the ten farmers in transitioning to strip cropping systems for the first time**

**From sole crops to strip cropping: decision rules of frontrunner farmers in the Netherlands**

Stella D. Juventia ^1*^, Dirk F. van Apeldoorn ^1,2,3^, Hilde Faber ^1,3,4^, Walter A. H. Rossing ^1^

^1^ Farming Systems Ecology Group, Wageningen University & Research, Wageningen, the Netherlands

^2^ Field Crops, Wageningen University & Research, Edelhertweg 10, Lelystad, the Netherlands

^3^ Centre for Crop Systems Analysis, Wageningen University & Research, Wageningen, the Netherlands

^4^ Land & Co, Costerweg, Wageningen, the Netherlands

**S3 Table. Main challenges mentioned by farmers (n = 10) in transitioning to strip cropping systems for the first time.** Challenges are organized per crop management phase and ordered in descending order of number of farmers’ responses. The challenges in bold were related to the strip cropping decisions that constitute farmers’ decision rules. The “D_”, “N_”, and “W_” in the abbreviations refer to the four synthetic topics “Spatio-temporal configuration design”, “Neighbor”, and “Strip Width”, respectively, which are used in the MFA analysis (section 2.3.2) and for the visualization of decision rules (S6 Table and S7 Fig).

| **Crop management phases** | **Challenges (n = number of responses)** | **Farmers’ strip cropping decisions (abbreviation)** |
| --- | --- | --- |
| Strategic planning | Knowledge on interactions, varieties, and mixtures (n = 10) |  |
|  | Labor demand (n = 8) |  |
|  | Market access (n = 5) |  |
|  | Timely field registration with the authorities by May 15 (n = 3) |  |
|  | **Field history in relation to crop rotation (n = 3)** | - Incorporate green manure crop before or during strip cropping implementation (D_design1) |
|  | Animal management in strips (n = 2) |  |
|  | **Crop choice in relation to inflexibility to react to yearly market changes (n = 1)** | - Widen the range of candidate crops by working with crop families instead of species (D_design2) |
| Soil preparation | Crop specific needs (n = 10) |  |
| Sowing | Knowledge on interactions, varieties, and mixtures (n = 10) |  |
|  | Crop specific needs (n = 10) |  |
|  | Limited machinery available from contract worker (n = 4) |  |
| Irrigation | Knowledge on interactions, varieties, and mixtures (n = 10) |  |
|  | **Crop specific needs (n = 10)** | - Reconsider crop choice when irrigation is not possible by e.g. selecting deep-rooted crops (W_wsfi1) - Do not spray, fertilize, or irrigate and accept potential yield loss (W_wsfi2) - Use current method/ machine, strip width irrelevant (W_wsfi3) |
|  | Labor demand (n = 8) |  |
|  | Damage to crop neighbor (n = 2) |  |
| Fertilization | Knowledge on interactions, varieties, and mixtures (n = 10) |  |
|  | **Crop specific needs (n = 10)** | - Do not spray, fertilize, or irrigate and accept potential yield loss (W_wsfi2) - Use current method/ machine, strip width irrelevant (W_wsfi3) |
|  | Labor demand (n = 8) |  |
|  | Limited machinery available from contract worker (n = 4) |  |
| Spraying | Knowledge on interactions, varieties, and mixtures (n = 10) |  |
|  | **Crop specific needs (n = 10)** | - Do not spray, fertilize, or irrigate and accept potential yield loss (W_wsfi2) - Use current method/ machine, strip width irrelevant (W_wsfi3) |
|  | **Damage to crop neighbor (n = 8)** | - Reduce between-row distance and/or increase strip width to increase space between strips (N_dmg1) - Drive carefully to reduce damage or residue carry-over to neighboring strip (N_dmg2) - Use section closure on spray boom (N_dmg3) - Keep between-row distance as monoculture (N_dmg4) |
|  | Limited machinery available from contract worker (n = 4) |  |
| Weeding | Knowledge on interactions, varieties, and mixtures (n = 10) |  |
|  | **Damage to crop neighbor (n = 8)** | - Reduce between-row distance and/or increase strip width to increase space between strips (N_dmg1) - Drive carefully to reduce damage or residue carry-over to neighboring strip (N_dmg2) - Use section closure on spray boom (N_dmg3) - Keep between-row distance as monoculture (N_dmg4) |
|  | **Trade-off between weed pressure, build-up of seedbank, and yield (n = 9)** | - Pre-crop to reduce weed pressure (W_ww1) - Mulch to suppress weed emergence (W_ww2) - Weed by e.g. mechanical means, herbicide spraying, flaming, hand weeding, and/or false seedbed (W_ww3) - Do not weed, accept potential yield loss (W_ww4) |
|  | Labor demand (n = 8) |  |
| Harvesting | Knowledge on interactions, varieties, and mixtures (n = 10) |  |
|  | **Crop specific needs (n = 10)** | - Use ‘upright’ varieties (N_bound1) - Trim off shoots outside strip boundary (N_bound2) - Hand-harvest produce outside strip boundary (N_bound3) - Sow crop neighbor that can be driven over or harvested earlier (N_avail1) - Adjust machine or buy new machine to harvest within the strip (N_avail2)^1^ - Rent machine to harvest within the strip (N_avail3) - Keep strip cropping plan (N_avail4) |
|  | Labor demand (n = 10) |  |
|  | Limited machinery available from contract worker (n = 4) |  |
|  | Communication with contract worker (n = 3) |  |
| All crop management phases | Practical experience (n = 9) |  |
|  | **Crop rotation in time and space (n = 9)** | - Incorporate green manure crop before or during strip cropping implementation (D_design1) - Widen the range of candidate crops by working with crop families instead of species (D_design2) |
|  | GPS technology, automatization and **adaptation of machines for strip cropping** **(n = 8)** | - Adjust or buy new machine (W_wa5) - Rent machine (W_wa6) |
|  | **Machine working width ≠ strip width (n = 5)** | - Use desired strip width, independent management per strip is possible (W_wa1) - Multiply strip width (W_wa2) - Install drip irrigation system (W_wa3) - Use section closure for spraying, fertilization, or irrigation (W_wa4) - Adjust or buy new machine (W_wa5) - Rent machine (W_wa6) - Spray, fertilize, or irrigate over part of the neighboring strips (W_wa7) - Use desired strip width, independent management per strip is possible but less efficient (W_wa8) - Accept inefficiency of over-spraying, -fertilization, or -irrigation of neighboring crops (W_wa9) |
|  | **Financing new technology (n = 5)** | - Adjust or buy new machine (W_wa5) - Rent machine (W_wa6) |
|  | Subsidy (n = 2) |  |
|  | Field scattering (n = 1) |  |
|  | Irregular field shape (n = 1) |  |

^1^ Two decisions grouped as one as they both present option for autonomy compared to renting machine from other actors, although they might incur very different financial cost
