# Supplementary material for: From sole crops to strip cropping: Decision rules of frontrunner farmers in The Netherlands
Source: PLoS One. 2025 Jul 24;20(7):e0329133. doi: 10.1371/journal.pone.0329133 (PMC12289020; doi:10.1371/journal.pone.0329133)
Supplement: S4 Table — (DOCX) [file pone.0329133.s004.docx]

**S4 Table: Farmers’ evaluation of the outcomes of their first year experimenting with strip cropping**

**From sole crops to strip cropping: decision rules of frontrunner farmers in the Netherlands**

Stella D. Juventia ^1*^, Dirk F. van Apeldoorn ^1,2,3^, Hilde Faber ^1,3,4^, Walter A. H. Rossing ^1^

^1^ Farming Systems Ecology Group, Wageningen University & Research, Wageningen, the Netherlands

^2^ Field Crops, Wageningen University & Research, Edelhertweg 10, Lelystad, the Netherlands

^3^ Centre for Crop Systems Analysis, Wageningen University & Research, Wageningen, the Netherlands

^4^ Land & Co, Costerweg, Wageningen, the Netherlands

**S4 Table. Farmers’ evaluation of the outcomes of their first year experimenting with strip cropping (n = 10).** Farmers qualitatively evaluated their strip cropping system compared to sole-crop monoculture in terms of positive, neutral, or negative outcomes. Indicators are organized per outcome category and ordered in descending order of farmers’ responses.

| **Outcomes** | **Indicators** | **Responses** |
| --- | --- | --- |
| Positive | Availability and feasibility of mechanization for strip cropping | 10 |
|  | Use of machines used in sole crop for strips | 7 |
|  | Aesthetic appeal | 7 |
|  | Size of insect population | 6 |
|  | Ease of strip cropping management | 6 |
|  | Input minimization (e.g. fertilizer, pesticide) | 5 |
|  | Pest and disease incidences | 5 |
|  | Market access as frontrunner farmer | 5 |
|  | Field bird population | 4 |
|  | Year-round green coverage | 3 |
|  | Soil compaction | 3 |
|  | Soil life | 3 |
|  | Risk of crop loss | 2 |
|  | Size of natural enemy population | 2 |
|  | Flexibility to respond to market changes | 1 |
| Neutral | Yield | 7 |
|  | Labor efficiency | 3 |
|  | Logistics when fields are clustered | 2 |
|  | Product price | 1 |
| Negative | Workload | 8 |
|  | Weed pressure | 5 |
|  | Labor cost | 4 |
|  | Product price | 3 |
|  | Pest and disease incidences | 2 |
|  | Size of natural enemy population | 1 |
|  | Labor efficiency | 1 |
