# Supplementary material for: From sole crops to strip cropping: Decision rules of frontrunner farmers in The Netherlands
Source: PLoS One. 2025 Jul 24;20(7):e0329133. doi: 10.1371/journal.pone.0329133 (PMC12289020; doi:10.1371/journal.pone.0329133)
Supplement: S5 Table — (DOCX) [file pone.0329133.s005.docx]

**S5 Table: An example of the changes in operational management decisions that farmer F1 made when implementing strip cropping systems compared to sole-crop monoculture**

**From sole crops to strip cropping: decision rules of frontrunner farmers in the Netherlands**

Stella D. Juventia ^1*^, Dirk F. van Apeldoorn ^1,2,3^, Hilde Faber ^1,3,4^, Walter A. H. Rossing ^1^

^1^ Farming Systems Ecology Group, Wageningen University & Research, Wageningen, the Netherlands

^2^ Field Crops, Wageningen University & Research, Edelhertweg 10, Lelystad, the Netherlands

^3^ Centre for Crop Systems Analysis, Wageningen University & Research, Wageningen, the Netherlands

^4^ Land & Co, Costerweg, Wageningen, the Netherlands

**S5 Table. An example of the changes in operational management decisions that farmer F1 made when implementing strip cropping systems compared to sole-crop monoculture.** The decisions were recorded per crop management phase and per crop.

| **Crop management phases** | **Crop-non-specific decisions** | **Crop-specific decisions** | | | | | |
| --- | --- | --- | --- | --- | --- | --- | --- |
|  |  | **Potato** | **Green Bean** | **Chicory** | **Grass—clover** | **Pumpkin** | **Cereal** |
|  | |  | | | | | |
| **Strategic planning** |  |  |  |  | • Widened strip width twice as wide to allow animal grazing | • Chose cultivar that does not produce many vines | • Widened strip width twice as wide to allow harvesting with combine |
| **Soil preparation** | • Invested in machinery that functions at right working width |  |  |  |  |  |  |
| **Fertilization** | • Invested in manure cart at right working width to enable crop specific fertilization in autumn  • Custom-built chicken mobile that is narrower than 3 m to allow fences around it and with front and back doors (not on sides) |  |  |  |  |  |  |
|  |  |  |  |  |  |  |  |
|  |  | **Potato** | **Green Bean** | **Chicory** | **Grass—clover** | **Pumpkin** | **Cereal** |
|  |  |  |  |  |  |  |  |
| **Sowing** | • Invested in 2 new sowing machines that match strip width and can sow all crops |  |  | • Invested in new sowing machines to sow more precisely |  |  |  |
| **Mechanical weeding** | • Invested in new camera-guided hoe.  • Used pigs against perennial weeds |  |  |  |  |  |  |
| **Irrigation** | NA |  |  |  |  |  |  |
| **Harvesting** |  | **•** Used conventional bunker harvester to avoid driving on neighboring strips by unloading at the headland. |  |  |  | • Could not use monoculture harvesting cart as it damaged neighbors  • Harvested in 2 stages: cut by hand and left in field, then picked up by ‘fruit-train’ | • Invested in combine with a working width equals strip width |
