# Supplementary material for: From sole crops to strip cropping: Decision rules of frontrunner farmers in The Netherlands
Source: PLoS One. 2025 Jul 24;20(7):e0329133. doi: 10.1371/journal.pone.0329133 (PMC12289020; doi:10.1371/journal.pone.0329133)
Supplement: S6 Table — (DOCX) [file pone.0329133.s006.docx]

**S6 Table: List of 49 decision rules comprising 11 conditions and 43 decisions formulated based on the coded interviews with the farmers**

**From sole crops to strip cropping: decision rules of frontrunner farmers in the Netherlands**

Stella D. Juventia ^1*^, Dirk F. van Apeldoorn ^1,2,3^, Hilde Faber ^1,3,4^, Walter A. H. Rossing ^1^

^1^ Farming Systems Ecology Group, Wageningen University & Research, Wageningen, the Netherlands

^2^ Field Crops, Wageningen University & Research, Edelhertweg 10, Lelystad, the Netherlands

^3^ Centre for Crop Systems Analysis, Wageningen University & Research, Wageningen, the Netherlands

^4^ Land & Co, Costerweg, Wageningen, the Netherlands

**S6 Table. List of 49 decision rules comprising 11 conditions and 43 decisions formulated based on the coded interviews with the farmers (n = 10).** The number of decision rules does not correspond with the total number of decisions because some decisions were related to several conditions (e.g. rent new machine), or because we pooled together different methods of crop management (e.g. different ways of weeding or irrigation) that were not necessarily tied to strip cropping systems. Responses indicate the number of farmers associated with each decision rule. The column describing codes in the decision tree shows how the decision rules are represented in the decision tree (S7 Fig). Nodes refer to the condition and decision that were used in the SCM; edges are not indicated here (see Fig 5).

| **Crop management phases** | **Decision rules** | | **Examples of relevant crops** | **Responses** | **Codes in the decision tree** | | | **Nodes (see Table 3 for explanation)** |
| --- | --- | --- | --- | --- | --- | --- | --- | --- |
| **Weeding, spraying** | IF the build-up of weed pressure the following years is a concern (e.g. as the following crop in rotation is susceptible to weed), THEN weed as frequent as in sole-crop monoculture, or plough | | Pumpkin, oats followed by onion | 4 | W_ww3 | | | Weed pressure,  Weeding methods |
|  | AND | |  |  |  | | |  |
|  | IF possible/desirable to make a false seedbed, THEN make a false seedbed either strip specific or the whole field | | Sugar beet | 1 | W_ww3 | | | Weed pressure,  Weeding methods |
|  | ELSE |  |  |  | |  |  |  |
|  | IF possible/desirable to spray herbicide for convenience, lower risk and cost, THEN spray herbicide as frequent as in sole-crop monoculture | | Cereal, onion | 1 | W_ww3 | | | Weed pressure,  Weeding methods |
|  | ELSE |  |  |  | |  |  |  |
|  | IF weed germination is even AND the crop at the neighboring strip will not catch fire, THEN do flame weeding e.g. 1-3 weeks after sowing or 2-5 weeks after crop emergence | | Onion, potato next to oat | 2 | W_ww3 | | | Weed pressure,  Weeding methods |
|  | ELSE | |  |  |  | | |  |
|  | IF possible to suppress the weed by mechanical weeding, THEN do mechanical weeding (e.g. hoe or harrow depending on planting distance) before the crop reach a certain height/canopy closure it is not possible to mechanically weed anymore or mow the annual weeds either strip specific or the whole field | | Maize and bean, onion, sugar beet, grass–clover | 6 | W_ww3 | | | Weed pressure,  Weeding methods |
|  | ELSE |  |  |  | |  |  |  |
|  | IF financial and/or human resources are sufficient to hire labors, THEN do hand weeding where necessary | | Onion, leek | 4 | W_ww3 | | | Weed pressure,  Resources,  Weeding methods |
|  | ELSE |  |  |  | |  |  |  |
|  | use mulch to suppress weed | | Chicory, oat-bean mixture destroyed and used as mulch for cauliflower | 1 | W_ww2 | | | Weed pressure,  Weeding methods |
|  | OR use pre-crop (e.g. luzerne, grass-clover) to clean the plot from weed | | Luzerne, grass-clover | 2 | W_ww1 | | | Weed pressure,  Weeding methods |
|  | OR do not weed and accept harvest loss | | Any crop | 2 | W_ww4 | | | Weed pressure,  Inefficiency,  Weeding methods |
| **Sowing, weeding, spraying,**  **harvesting** | IF between-strip distance is wide enough (e.g. not to damage the neighboring crops), THEN keep the between-row distance as in sole-crop monoculture | | Any crop | 2 | N_dmg4 | | | Crop neighbor damage,  Between-row distance adjustment |
|  | ELSE |  |  |  | |  |  |  |
|  | IF the neighboring crops are not sown yet, THEN spray as usual without concern that the spray (residue) will be blown over and negatively affect the neighboring crops | | Barley next to maize and hemp | 2 | N_dmg2 | | | Crop neighbor damage,  Timing |
|  | ELSE |  |  |  | |  |  |  |
|  | increase the strip width (e.g. from 3.15 m to 3.20 m) and/or reduce the between-row distance (e.g. from 50 cm to 46 cm) | | Wheat and sugar beet, cabbage and onion | 1 | N_dmg1 | | | Crop neighbor damage,  Strip width adjustment,  Between-row distance adjustment |
|  | OR in case of spraying, to minimize problems with spraying residue: account for weather or close sections | | Wheat and sugar beet, cabbage and onion | 3 | N_dmg3 | | | Crop neighbor damage,  Machine investment |
|  | OR, IF current (own) machine modification is possible AND/OR financial means are sufficient, THEN modify current (own) machine | | Onion next to bean | 1 | W_wa5 | | | Crop neighbor damage,  Resources,  Machine investment |
|  | ELSE | |  |  |  | | |  |
|  | use current (own) machine, drive carefully and accept either lower weeding/spraying quality/efficiency at the strip edges or the potential damage in the crop at the neighboring strip | | Cabbage or celeriac next to beans, cereal next to cabbage | 2 | N_dmg2 | | | Crop neighbor damage,  Resources,  Inefficiency,  Machine investment |
| **Sowing, weeding, spraying,**  **harvesting** | IF it is possible to standardize (a) between-row distance(s) that is/are common for various crops and machines for various crop management phases, THEN modify and choose (a) common between-row distance(s) e.g. 25 cm for cereals, 50 or 75 cm or 46 cm and 75 cm for (root) crops without and with ridges respectively | | Oats, onion with 46 cm between-row distance instead of the common 30 cm | 3 | W_wa5 | | | Between-row distance adjustment |
|  | ELSE |  |  |  | |  |  |  |
|  | choose (a) between-row distance(s) common to most/ most frequently used machines, modifying the other machines or the between-row distance for the other crops | | Any crop | 1 | W_wa5 | | | Between-row distance adjustment |
| **Harvesting** | IF strip width equals harvester width AND neighboring strip is not required to unload and store the harvest, THEN keep strip cropping plan using current machine as in sole-crop monoculture | | Leek, cereals, bean, potato, onion, alfalfa | 6 | N_avail4 | | | Working width,  Machine investment |
|  | ELSE |  |  |  | |  |  |  |
|  | IF current (own) machine modification is possible AND/OR financial means are sufficient, THEN either adjust current harvester or buy a new machine to harvest and load within the strip e.g. a bunker harvester with sufficient storage volume | | Sugar beet, cabbage, pumpkin, potato, chicory, onion, carrot harvested by potato harvester | 4 | N_avail2 | | | Working width,  Resources,  Machine invest |
|  | ELSE |  |  |  | |  |  |  |
|  | IF possible to rent from other actors, THEN rent from other actors to harvest and load within one strip with e.g. bunker harvester | | Sugar beet, potato | 2 | N_avail3 | | | Working width,  Resources,  Machine investment |
|  | ELSE |  |  |  | |  |  |  |
| **Strategic planning, harvesting** | sow a neighboring crop that either can be driven over (e.g. green manure, grass–clover) or a crop with earlier harvest date | | Parsnip or carrot with grass–clover as neighbor; broccoli, celeriac, or potato with a mixture of oats, clover, vetch and phacelia as neighbor; sugar beet with a cereal as neighbor | 3 | N_avail1 | | | Working width,  Crop choice adjustment |
| **Fertilization** | IF the same dose or type of fertilization can be applied to all crops (i.e. basic fertilization), THEN fertilize full-field with current (own) machinery as in sole-crop monoculture | | Any crop | 4 | W_wsfi3 | | | Working width,  Machine investment |
|  | ELSE |  |  |  | |  |  |  |
|  | IF machine width equals or is smaller but a factor of the strip width, THEN use desired strip width. Calculate the crop-specific fertilizer need, considering the pre-crop and potential leaching with irrigation, fertilize specific strips. This can be in the form of additional fertilization (e.g. chicken pellet) for crops with higher nutrients demand. | | Cauliflower, leek, spinach; maize and cabbage (a lot); and chicory (nothing) | 9 | W_wa1 | | | Working width,  Strip width adjustment |
|  | OR, IF machine width is smaller than strip width but slightly wider than half of the strip width, THEN shift a little bit while driving and accept that the middle of the strip will also get an extra fertilization | | Maize and hemp | 1 | W_wa8 | | | Working width,  Inefficiency,  Machine investment |
|  | ELSE |  |  |  | |  |  |  |
|  | IF current (own) machine modification/renting from other actors is possible, THEN rent from other actors | | Any crop | 5 | W_wa6 | | | Working width,  Resources,  Machine investment |
|  | ELSE |  |  |  | |  |  |  |
|  | modify the current (own) machine (e.g. pneumatic fertilizer spreader), driving in between two strips | | Broccoli, pumpkin | 5 | W_wa5 | | | Working width,  Resources,  Machine investment |
|  | OR use section closure (i.e. closing specific nozzles) to fertilize specific crops is an option | | Any crop | 2 | W_wa4 | | | Working width,  Resources,  Machine investment |
|  | OR accept that the neighboring strip will also get an extra fertilization | | Any crop | 4 | W_wa7 | | | Working width,  Resources,  Inefficiency,  Machine investment |
| **Irrigation, spraying** | IF the same dose can be applied to all crops, THEN use current (own) method/machine (e.g. sprinkler, canon) as in sole-crop monoculture | | Any crop | 4 | W_wsfi3 | | | Working width,  Machine investment |
|  | ELSE |  |  |  | |  |  |  |
|  | IF the current (own) machine width equals a multiple of the strip width, THEN calculate the crop-specific need and irrigate/spray specific strips. Section closure (i.e. closing specific nozzles) to irrigate/spray specific crops is an option | | Cereal, cabbage, carrot | 3 | W_wa1, W_wa4 | | | Working width,  Machine investment |
|  | OR, IF the current (own) machine width does not equal a multiple of the strip width, THEN modify current (own) machine or rent or buy a new machine with equal or slightly wider width | | Any crop | 7 | W_wa5, W_wa6 | | | Working width,  Machine investment |
|  | OR, IF the crop-specific need requires higher precision (e.g. irrigate 10% of surface area just before sowing or after germination), THEN use a tank (e.g. 1000, 5000 liter) to irrigate/spray a specific strip, precisely applying over 5-cm width on each row | | Carrot, parsnip, chicory, cabbage and onion | 2 | W_wa3 | | | Crop specific needs,  Machine investment |
|  | ELSE |  |  |  | |  |  |  |
|  | IF economy is not a challenge, THEN install a drip irrigation system with higher efficiency than any other systems and can be applied both in strip cropping and sole-crop monoculture and accept that hoeing might be more difficult | | Any crop | 3 | W_wa3 | | | Working width,  Resources,  Inefficiency,  Machine investment |
|  | ELSE |  |  |  | |  |  |  |
|  | IF modification of current (own) machine is not possible BUT irrigation/spraying is essential for the survival of the cash crops, THEN use current (own) (e.g. sprinkler, canon) as in sole-crop monoculture | | All crops and too much for pumpkin | 4 | W_wa7 | | | Resources,  Crop specific needs,  Machine investment |
|  | OR accept (unnecessary) over-irrigation/spraying for some crops | | All crops and too much for pumpkin | 4 | W_wa9 | | | Resources,  Crop specific needs,  Inefficiency  Machine investment |
|  | ELSE |  |  |  | |  |  |  |
|  | choose deep-rooted crops which can better make use of soil moisture especially for germination or use resistant varieties | | Potato (Vitabella cultivar) | 2 | W_wsfi1 | | | Resources,  Crop specific needs,  Crop choice adjustment |
|  | OR do not irrigate/spray and accept some yield loss | | Chicory, cereal, maize | 2 | W_wsfi2 | | | Resources,  Crop specific needs,  Machine investment |
| **Sowing, weeding, harvesting** | IF machine width equals the strip width, THEN use current (own) machine | | Any crop | 6 | W_wa1 | | | Working width,  Machine investment |
|  | ELSE |  |  |  | |  |  |  |
|  | IF machine width is smaller than the strip width, THEN use current (own) machine, going over the same strip several times despite lower efficiency than if machine width was to match strip width | | Any crop | 5 | W_wa8 | | | Working width,  Inefficiency,  Machine investment |
|  | ELSE/OR |  |  |  | |  |  |  |
|  | IF current (own) machine modification is possible AND/OR financial means are sufficient, THEN either adjust current machine or buy a new machine, ideally with a working width common to all relevant crop management phases | | Any crop | 3 | W_wa5 | | | Working width,  Resources,  Machine investment |
|  | ELSE |  |  |  | |  |  |  |
|  | IF possible to rent machine whose width equals strip width from other actors, THEN rent from other actors | | Any crop | 5 | W_wa6 | | | Working width,  Resources,  Machine investment |
|  | ELSE |  |  |  | |  |  |  |
|  | double (or triple etc.) the strip width to fit current machine width | | Cereal | 1 | W_wa2 | | | Working width,  Resources,  Strip width adjustment |
| **Strategic planning** | IF all the crops in the first year of strip cropping application can follow the preceding crop before strip cropping application based on agro-ecological reasonings, THEN keep the crop choice and rotation plan | | Any crop | 0 | D_design3 | | | Rotation,  Crop choice adjustment |
|  | ELSE |  |  |  | |  |  |  |
|  | either sow perennial grass–clover or replace the crops that should not directly follow the preceding crop with a break crop that fits in the rotation | | Onion replaced by leek; potato followed by cereal instead of potato followed by parsnip | 2 | D_design1 | | | Rotation,  Crop choice adjustment |
|  | OR work with crop families instead of species to widen the range of candidate crops | | Any crop | 2 | D_design2 | | | Rotation,  Crop choice adjustment |
| **Spraying** | IF exploring the potential of biological control from the strip cropping implementation is not an objective, THEN spray as usual | | Any crop | 0 | O_bb6 | | | Biodiversity/ biocontrol,  Spraying intensity |
|  | OR |  |  |  | |  |  |  |
| **Planning, spraying** | IF frequent spraying poses a logistical/managerial challenge in the first year of strip cropping application, THEN consider replacing the crop(s) that require(s) frequent spraying with those that do not, but would fit in the crop rotation | | Potato replaced by cereal | 1 | O_bb3 | | | Biodiversity/ biocontrol,  Rotation,  Crop choice adjustment |
|  | ELSE |  |  |  | |  |  |  |
| **Spraying** | use a non-resistant variety (with better taste) to test strip cropping potential | | Cauliflower, cabbage, potato | 1 | O_bb4 | | | Biodiversity/ biocontrol,  Rotation,  Crop choice adjustment |
|  | OR spray less compared to sole-crop monoculture | | Cauliflower, cabbage, potato | 5 | O_bb5 | | | Biodiversity/ biocontrol,  Rotation,  Spraying intensity |
|  | AND/OR |  |  |  | |  |  |  |
| **Sowing, spraying** | IF exploring the potential of biodiversity from the strip cropping implementation is an objective, THEN do not spray (e.g. tracer that kills not only the thrips but also other insects and natural enemies) | | Any crop | 6 | O_bb5 | | | Biodiversity/ biocontrol,  Rotation,  Spraying intensity |
|  | OR consider sowing cereal-flower mix at the cereal strip or field border, sacrificing a whole strip for a beetle bank sacrificing a whole field as semi-natural habitat for birds, or having hedges as semi-natural habitat around the field | | Phacelia next to onion for trips control; sugar beet; cereal, flower, and perennial comfrey | 5 | O_bb2 | | | Biodiversity/ biocontrol,  Rotation,  Semi-natural elements |
|  | BUT | |  |  |  | | |  |
| **Spraying** | IF pest pressure is considered too high that it will cause significant crop damage, THEN slightly delay the spraying ideally after observing the potential strip cropping effect (if any) but before the damage level is too high | | Cabbage, onion | 3 | O_bb1 | | | Biodiversity/ biocontrol,  Rotation,  Timing |
| **Irrigation, spraying, harvesting** | IF soil compaction is not a concern, THEN use current (own) machine as in sole-crop monoculture | | Any crop | 0 | O_soil6 | | | Soil compaction,  Machine investment |
|  | ELSE |  |  |  | |  |  |  |
|  | IF financial means are sufficient, THEN cover them by grass–clover or flower mixture to allow extra driving over without compacting the soil or damaging the crops on the edge rows | | Any crop | 2 | O_soil1 | | | Soil compaction,  Resources,  Green cover |
|  | OR install and use fixed traffic lanes to not compact the cultivated soil | | Any crop | 1 | O_soil2 | | | Soil compaction,  Resources,  Machine investment |
|  | ELSE |  |  |  | |  |  |  |
|  | either use a smaller and lighter machine with narrower width even if it means going over the same strip several times | | Any crop | 1 | O_soil3 | | | Soil compaction,  Inefficiency,  Machine investment |
|  | OR drive less frequently | | Any crop | 1 | O_soil4 | | | Soil compaction,  Timing |
|  | OR for harvesting, drive a tipper on neighboring strip to unload and store some of the harvest | | Any crop | 3 | O_soil5 | | | Soil compaction,  Machine investment |
| **Harvesting** | IF a crop grows within the strip boundary, THEN use current (own) machine as in sole-crop monoculture | | Any crop | 0 | N_bound4 | | | Crop neighbor damage,  Machine investment |
|  | ELSE |  |  |  | |  |  |  |
|  | IF there are varieties that grow upright, THEN use upright varieties (e.g. pumpkin variety Butter nut instead of Orange summer or Big banana) | | Pumpkin | 2 | N_bound1 | | | Crop neighbor damage,  Crop choice adjustment |
|  | ELSE |  |  |  | |  |  |  |
|  | IF current (own) machine modification is possible, THEN adjust current (own) machine to trim the vines growing outside the strip boundary | | Pumpkin | 1 | N_bound2 | | | Crop neighbor damage,  Resources,  Machine investment |
|  | ELSE |  |  |  | |  |  |  |
|  | hand-harvest produce that are growing outside the strip boundary | | Pumpkin | 1 | N_bound3 | | | Crop neighbor damage,  Resources,  Machine investment |
