# Supplementary material for: From sole crops to strip cropping: Decision rules of frontrunner farmers in The Netherlands
Source: PLoS One. 2025 Jul 24;20(7):e0329133. doi: 10.1371/journal.pone.0329133 (PMC12289020; doi:10.1371/journal.pone.0329133)
Supplement: S7 Fig — (DOCX) [file pone.0329133.s007.docx]

**S7 Fig: Decision tree visualizing farmers’ decision rules**

**From sole crops to strip cropping: decision rules of frontrunner farmers in the Netherlands**

Stella D. Juventia ^1*^, Dirk F. van Apeldoorn ^1,2,3^, Hilde Faber ^1,3,4^, Walter A. H. Rossing ^1^

^1^ Farming Systems Ecology Group, Wageningen University & Research, Wageningen, the Netherlands

^2^ Field Crops, Wageningen University & Research, Edelhertweg 10, Lelystad, the Netherlands

^3^ Centre for Crop Systems Analysis, Wageningen University & Research, Wageningen, the Netherlands

^4^ Land & Co, Costerweg, Wageningen, the Netherlands

**S7 Fig. Decision tree visualizing farmers’ decision rules.** Columns in the diagram represent the four dimensions of diversity: time, genes, space, and operational crop management. The rotated text on the left side of the tree shows the four synthetic topics (Objectives, Spatio-temporal configuration design, Crop neighbors, and Strip width) and the eight management phases (strategic planning, soil preparation, sowing/planting, fertilization, irrigation, spraying, weeding, and harvesting). Farmers’ decision rules and farmer codes are presented in green and orange boxes. Blue boxes represent decision rules proposed by agronomists. Dotted arrows represent feedback loops.

<https://lucid.app/publicSegments/view/f060e9eb-6a4c-4868-b37d-7cb63abf5218/image.jpeg>
