# Supplementary material for: From sole crops to strip cropping: Decision rules of frontrunner farmers in The Netherlands
Source: PLoS One. 2025 Jul 24;20(7):e0329133. doi: 10.1371/journal.pone.0329133 (PMC12289020; doi:10.1371/journal.pone.0329133)

**S8 Fig: Cluster plot from fuzzy clustering of the ten interviewed farmers**

**From sole crops to strip cropping: decision rules of frontrunner farmers in the Netherlands**

Stella D. Juventia ^1*^, Dirk F. van Apeldoorn ^1,2,3^, Hilde Faber ^1,3,4^, Walter A. H. Rossing ^1^

^1^ Farming Systems Ecology Group, Wageningen University & Research, Wageningen, the Netherlands

^2^ Field Crops, Wageningen University & Research, Edelhertweg 10, Lelystad, the Netherlands

^3^ Centre for Crop Systems Analysis, Wageningen University & Research, Wageningen, the Netherlands

^4^ Land & Co, Costerweg, Wageningen, the Netherlands

**S8 Fig. Cluster plot from fuzzy clustering of the ten interviewed farmers.** The three clusters are embedded within the 95% confidence ellipse of cluster 3, indicating relatively high level of fuzziness were observed (normalized Dunn’s partition coefficient = 0.07). Centroid of all the clusters was close to the origin of the axes, indicating that there was no distinguishing relation that could explain cluster formation.


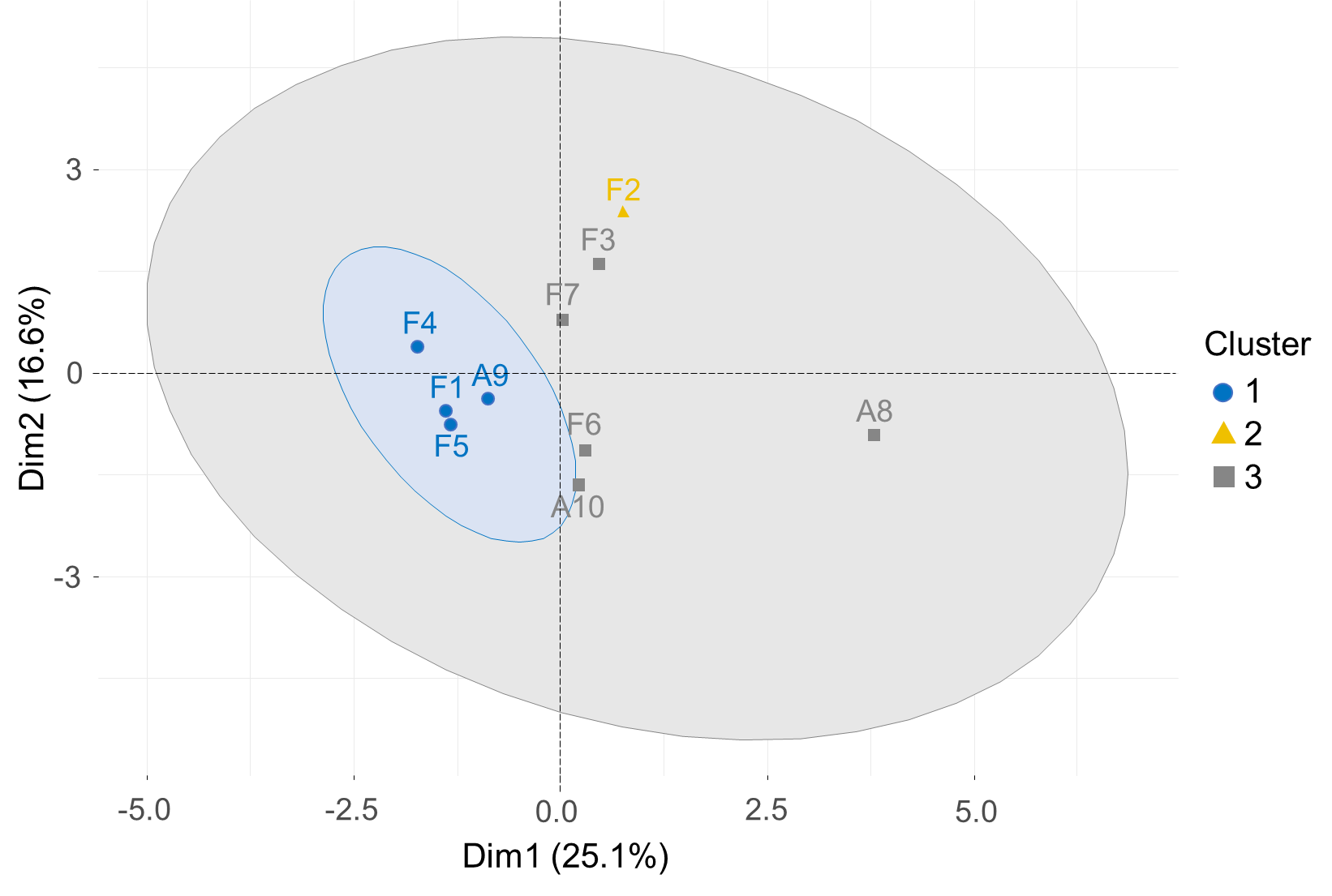

Supplement: S8 Fig — (DOCX) [file pone.0329133.s008.docx]
